# Motion sequencing reveals hidden patterns of repetitive behavior in a mouse model of epilepsy

**DOI:** 10.64898/2025.12.04.692371

**Published:** 2025-12-09

**Authors:** Jennifer Koehler, Quinn Harvey, Olivia Hoffmann, Jose Ezekiel Clemente Espina, Barry A. Schoenike, Grant Weiss, Jamie L. Maguire, Avtar S. Roopra

**Affiliations:** 1 Department of Neuroscience, University of Wisconsin-Madison School of Medicine and Public Health, Madison, WI 53705, USA; 2 Molecular and Cellular Pharmacology Graduate Program, University of Wisconsin-Madison School of Medicine and Public Health, Madison, WI 53705, USA; 3 Cellular and Molecular Biology Graduate Program, University of Wisconsin-Madison School of Medicine and Public Health, Madison, WI 53705, USA; 4 Department of Neuroscience, Tufts University School of Medicine, Boston, MA 02111, USA; 5 Department of Pharmacology & Chemical Biology, Emory University School of Medicine, Atlanta, GA 30322, USA

## Abstract

Epilepsy is the 4th most prevalent neurological condition with 50 million cases worldwide. Patients with epilepsy bare a disproportionate burden of cognitive decline and psychiatric disorders which remain poorly understood and go underdressed by current anti-epileptic treatments. Furthermore, pre-clinical work on behavioral comorbidities can be hampered by current testing frameworks which rely on well-defined, discreet tests with limited repeatability. Recent work has demonstrated a role for machine learning modalities such as Motion Sequencing (MoSeq) in assessing behavioral differences between naive and epileptic. In this study we combined MoSeq with a novel analysis pipeline to uncover repetitive behaviors in chronically epileptic mice. These repetitive behaviors emerge alongside epilepsy specific racing behaviors which persist in epileptic mice as disease progresses. Finally, we show that epileptic mice have more fragile and dispersed behavioral networks. Together, these results lay a groundwork for extracting clinically relevant phenotypes from MoSeq data throughout disease progression.

## Introduction

Epilepsy is the 4^th^ most common neurological condition affecting over 65 million people worldwide(1). Beyond seizures, more than 50% of people with epilepsy experience at least one psychiatric comorbidity(2,3) including depression, anxiety, obsessive compulsive disorder (OCD), and attention deficit hyperactivity disorder (ADHD)(4–9). These comorbidities result in reduced quality of life and often go unaddressed by current anti-seizure medications(6,10–12). There is a lack of consensus regarding the relationship between behavioral comorbidities and seizures. One school of thought proposes that seizures drive the emergence of comorbidities whereas another hypothesizes that an underlying mechanism drives both(2,13,14).

Having psychiatric comorbidities is associated with both more severe epilepsy and drug refractory disease(15,16). The mechanism driving this association is largely unknown(17). However, there are several factors associated with both psychiatric disorders and epilepsy including but not limited to: hypothalamic-pituitary-adrenal (HPA) axis dysfunction, dysregulated neurotransmitters, and inflammatory mechanisms(2,18). It is possible that in most cases, a combination of these factors contributes to the emergence of a given comorbidity as well as the development of disease. Indeed, recent work has shown that activation of the HPA axis during early life stress has a sex dependent effect on sudden unexpected death in epilepsy (SUDEP)(19).

Current tools available to analyze behavioral comorbidities rely on discrete tests, such as the marble burying test for compulsive burying and Go-no Go test for impulsive behavior, which have well defined but limited outcome measures. Furthermore, certain behavioral tests risk carry-over effects when repeated too often in close succession(20). In contrast, Motion Sequencing (MoSeq) is a machine learning platform combined with 3D video analysis, that finds behavioral differences between epileptic and non-epileptic mice at a frame-by-frame rate(30–34) in an unbiased manner. MoSeq divides behavior into sub-second modules of behavior, known as “syllables,” comprised of activities such as a “scrunch” or “rear-up”. Others have used rates of syllable usage to distinguish epileptic and naïve mice as well as identify seizure behavior during interictal periods(25). However, while each syllable on its own has little clinical relevance pertaining to specific behavioral comorbidities, we reasoned that when sequenced in chronological order behavioral motif may hold greater clinical significance. From these sequences we can characterize the frequency of transitions, or “grammar”, between behavioral modules in epileptic and non-epileptic mice. We combined MoSeq with a novel workflow based on analyzing concatenated syllables sequences and network theory. This analysis reveals the emergence of an OCD like perseverative behavior in the kainic acid mouse model of epilepsy. We also find a hyperactivity phenotype associated with epilepsy specific modules of behavior that emerge and persist throughout chronic disease. Further, we quantified the emergence of epilepsy specific sedentary behaviors during disease progression. Finally, we show how epilepsy leads to perturbed and increasingly fragile behavioral networks in chronic disease. Together, this analysis provides a novel framework for analyzing MoSeq data which reveals clinically relevant behavioral phenotypes.

## Methods

### Systemic kainic acid model

FVB/Nhsd mice were weighed and singly housed in observation chambers for the duration of the injections. Mice were injected intraperitoneally (i.p.) with synthetic kainic acid (KA) (7.5mg/kg for FVB) dissolved in 0.9% saline (#7065, Tocris Bioscience, Bristol, United Kingdom). At twenty-minute intervals, mice were given 7.5 mg/kg injections of KA up to the third injection; the dosage was then reduced to 5.0mg/kg. Animals continued to receive 5.0 mg/kg KA every twenty minutes for up to 10 injections. If an animal experienced two or more Class V or VI seizures within a single twenty-minute cycle, the subsequent KA injection was skipped. Injections resumed for the next round unless the animal reached status epilepticus (SE), and animals were considered to have reached SE after experiencing at least five Class V or VI seizures within a 90-minute window. During induction of SE, seizures were scored using a modified Racine Scale (86) where I = freezing, behavioral arrest, staring spells; II = head nodding and facial twitches; III = forelimb clonus, whole-body jerks or twitches; IV = rearing; V = rearing and falling; VI = violent running or jumping behavior. KA mice were observed for 1–2 hours after SE was achieved. Animals were returned to their home cages post SE. In the days following injection, animals were weighed and injected with 0.9% saline (s.c.) if body weight decreased by more than 0.5g and given gel diet (cat #) to aid in recovery.

### Behavioral Seizure recording.

To verify that mice had developed epilepsy, all 12- and 20-weeks post-SE mice were video recorded for behavioral seizures. Male and female FVB mice were singly housed in observation chambers for the duration of recording with access to food and water, and mice that did not exhibit at least 1 seizure per week (0.2/day) during the baseline recording period (or who died during recording) were triaged from the analysis. Videos were then reviewed and scored by an experimenter using the modified Racine scale, and only class IV-VI were recorded.

### Behavioral data acquisition

Behavioral data acquisition was performed similar to previous descriptions(21,23,25). In brief, mice were placed in an open arena (17 inch diameter and 16 inch walls, 14317, United States Plastics Corp) and recorded immediately after. The opaque enclosure was sandpapered (4664A16, McMaster-Carr) and spray painted black (54TG81, Krylon Industrial) to avoid image artifacts. The enclosure was cleaned between mice with 70% ethanol. Each animal was allowed to freely explore the enclosure for 20 min. For disease epilepsy progression experiments, the mice were recorded at the same time every day during the light cycle at 1.5-, 12-, and 20 after the inductions of status epilepticus. The recordings occurred during the interictal period, and no mice were observed to have behavioral seizures prior to placement in the recording enclosure.

### Behavioral recording

Data acquisition was performed as previously described(21,23,25). Briefly, each mouse was tracked in 3D using a Kinect 2 camera suspended .65 cm over the arena providing a top-down view. Raw data from the camera was then sent to the acquisition and analysis computer (Ryzen 7 5800X AMD 8-core with 16GB DDR4 RAM and EVGA GeForce RTX 3070 Ti FTW3 8GB graphics card) via USB 3.0 cables and depth frames were retrieved at 30 frames per sec

### Motion Sequencing (MoSeq) Data extraction

The data preprocessing, extraction and modeling pipeline was performed as previously described(21,23,25). In brief, software was used to track the mouse in the enclosure and extract is position, orientation, height, size, and shape from the depth data. The 3D image of the mouse was extracted from that data using a previously described pipeline(21,23).

### MoSeq Data analysis – Transition Matrices

For each mouse we recorded the frequency with which one syllable transitioned into the next: thre frequency of “incoming syllable’ -> ‘outgoing syllable’. This data was used to generate a n x n transition matrix where n = number syllables which explain 99% of behavioral variance (see code).

### Network analysis – transition matrices

Individual non-normalized transition matrices were labeled to have “incoming_syllable number” along the side, and “outgoing_syllable number” along the top. Our individual non-normalized transition matrices match the structure of the matrices generated by MoSeq. Prior to network analysis we transformed transition matrices into a long format, with one column reserved for incoming syllable, another column for outgoing syllable, and a third column for the transition frequency (i.e. the number of transitions for that incoming outgoing pair of syllables). This output was then used to generate directed networks in cytoscape for each individual naïve and epileptic mouse at each timepoint. In these networks the nodes represented the syllables, and the edge represented a transition from one syllable to another, weighted by frequency. We also generated the same networks using the Python networkx library(26). The following network measures were collected: edge count, average neighbor degree, average degree connectivity, closeness vitality, betweenness centrality, and closeness centrality. Each metric was analyzed for significance using a Mann Whittney test. For Edge count at 12 weeks post SE, we also compared the first quartile and robust max (defined as the top three values).

### Edge count

Edge count is the total number of edges for each node. We calculated edge count in two different ways. For figure 6 and supplemental we used weighted edges, each edge representing a transition with the weight associated with the transition frequency in numbers. For figure 3, we replicated the edges for each node pairs by the number of transitions that edge pair had (for example, instead of incoming 2 outgoing 4 having one edge with a weight of 20, there would now be 20 edges all with a weight of 1).

### Average neighbor degree

Average neighbor degree is the average weighted edge count $\left(s_{i}\right)$ for the neighbors of a given. Since we used directed networks, the neighborhood $\left(N_{\left(i\right)}\right)$ of a node is determined based on incoming and outgoing nodes $\left(j\right)$ of a given node $\left(i\right)$, considering the weighted edge $\left(w_{ij}\right)$ that links nodes i and j times the edge count $\left(k_{j}\right)$ of nodes j. This can be calculated for weighted networks using the following formula:

$$k_{nn,j}^{w}=\frac{1}{s_{i}}\sum_{j\in N_{\left(i\right)}}w_{ij}k_{j}$$

Average neighbor degree measures the connectivity of a network by measuring the edge count of the neighbors of a given node, higher average neighbor degree indicates increased network connectivity and reduced node dispersion. Since average neighbor degree does not consider node number for different networks, it cannot be compared across timepoints that have a different max number of nodes for their respective networks.

### Average degree connectivity

Average degree connectivity is average weighted edge count of all neighboring nodes $\left(N_{\left(i\right)}\right)$ surrounding a node i of a given weighted edge count $\left(s_{i}\right)$. The weighted edge $\left(w_{ij}\right)$ that links the node of interest $\left(i\right)$ to its neighboring nodes $\left(j\right)$ is multiplied by the total edge count $\left(k_{j}\right)$. Average degree connectivity can be calculated using the following formula:

$$k_{n,nj}^{w}=\frac{1}{s_{i}}\sum_{j\in N_{\left(i\right)}}w_{ij}k_{j}$$

Average degree connectivity measures the connectivity of a network via the connectivity of nodes with a given edge count, networks with higher average degree connectivity are less dispersed and more connected.

### Closeness vitality

The closeness vitality of a node is the change in the sum of distances between all nodes when said node is removed from the network. This can be determined by calculating the wiener index of the graph with the node and without the node. The formula for the wiener index is below:

$$W\left(G\right)=\sum_{\left\{u,v\right\}\subseteq V\left(G\right)}d\left(u,v\right)$$

Wherein G represents the network, $V\left(G\right)$ is the set of vertices in a graph, or nodes, and $d\left(u,v\right)$ is the distance of the shortest path connecting the nodes. By measuring closeness vitality we can assess how vulnerable a network is to perturbations.

### Betweenness Centrality

Betweenness centrality measures the dependance of the network on a given node $\left(v\right)$ by summing the fraction of node pairs shortest paths $\left(s,t\right)$ that go through said node. The formula is displayed below:

$$c_{B}\left(v\right)=\sum_{s,t\in V}\frac{\sigma \left(s,t\left|v\right.\right)}{\sigma \left(s,t\right)}$$

Were v is a set of nodes and $\sigma \left(s,t\right)$ is the number of shortest paths, $\sigma \left(s,t\left|v\right.\right)$ is the number of shortest paths passing through the node $\left(v\right)$ of interest. V cannot be s or t. By measuring betweenness centrality we can assess the importance of a given node to information flow in a network and wholistically the extent to which hub nodes exist within a network. Since this formula accounts for the total number of nodes in a network, it can be compared across different timepoints in epilepsy progression which also have different max node numbers for their networks.

### Closeness Centrality

The closeness centrality of a given node in a directed graph represents the independent centrality of a given node, by calculating the reciprocal of the average shortest incoming path to node $\left(u\right)$ oval all (𝑛 − 1) reachable nodes. The formula for closeness centrality is listed below:

$$C\left(u\right)=\frac{n-1}{\sum_{v=1}^{n-1}d\left(v,u\right)}$$

Wherein $d\left(v,u\right)$ is the shortest path distance between nodes v and u. Closeness centrality allows us to measure how dispersed the nodes of a network are separate from edge counts.

### Word clouds.

Word clouds were generated using wordclouds.com. The size of the word was based on the number of times a given behavior was repeated in the syllable list. For instance, if ‘edge racing’ was repeated twice it would have a size of 2. See supplemental data for the syllable lists that generated the word clouds.

### Syllable sequences.

The sequence of all syllables that a mouse moves through in the recording period underlies the generation of transition matrices. We extracted syllable sequences for each mouse and recorded bouts of repetitive behavior.

Following syllable extraction from MoSeq, we quantified repetitive versus non-repetitive behavioral alternations using a sliding three-syllable window. Let the MoSeq-derived syllable sequence for a given animal be:

$${\text{S}}_{\text{1}}\text{,}\;{\text{S}}_{\text{2}}\text{,}...\text{ST,}$$

where each s_t_ denotes the integer-encoded syllable at time t. For each overlapping triplet

$$\left({\text{S}}_{\text{t}}\text{,}\;{\text{S}}_{\text{t+1}}\;{\text{S}}_{\text{t+2}}\right)\text{,}\;\text{where}\;\text{t=1,}...\text{T-2,}$$

we classified the pattern as non-repetitive if the first and third syllables differed, and repetitive if they were identical. We formalized this using a binary indicator variable

$${\text{b}}_{\text{t}}\text{=1}\;\text{if}\;{\text{S}}_{\text{t}}\neq {\text{S}}_{\text{t+2}}\;\text{and}\;\text{0}\;\text{if}\;{\text{S}}_{\text{t}}{\text{=S}}_{\text{t+2}.}$$

Thus, an example non-repetitive alternation such as 5,6,9 yields b_t_ = 1, whereas a repetitive alternation such as 12,1,12 yields b_t_ = 0. This procedure generates a binary sequence b_1_,…,b_T−2_ summarizing the alternation structure of the entire behavioral session.

To quantify overall behavioral flexibility, we calculated the fraction of non-repetitive alternations as:

$$f=\frac{1}{T-2}\sum_{t-1}^{T-2}bt$$

To assess the persistence of repetitive or flexible patterns, we next quantified the lengths of consecutive non-repetitive versus repetitive alternation bouts. Within the binary sequence b_t_, we defined a run as any maximal contiguous block of constant value (all 1s or all 0s). If a run begins at index s_k_ and ends at s_k+1_-1, its length is:

$${\text{L}}_{\text{k}}\text{=}\;{\text{S}}_{\text{k+1}}{\text{-S}}_{\text{k}}$$

These run lengths were used to compute summary statistics for each animal and condition.

### Statistical analysis

For all statistical analysis—unless otherwise specified—*q* < 0.05 or *P* < 0.05 corrected for multiple comparisons was considered statistically significant (*P/*q* < 0.05, **P/*q* < 0.01, ***P/*q* < 10^−3^, and ****P/*q* < 10^−4^). Data were graphed as means ± SD in Prism 10 software (GraphPad Software, La Jolla, CA). Statistical tests were also performed in Prism. Syllable usage, network analysis, and sequence analysis were analyzed by *t* test, two-way ANOVA/mixed-effects models, or one-way ANOVA, with Tukey’s correction for multiple comparisons or the two-stage step-up method of Benjamini, Krieger, and Yekutieli (abbreviated BKY in figure legends). Kruskal-Wallis tests with Benjamini, Krieger, Yekutieli correction for data with >2 conditions. Kolmogorov-Smirnov tests were used to test the differences in distributions. Fisher’s exact test was used for measuring the participation in behavioral tests with p-value reported. All statistical tests were two-tailed.

## Results

### Epileptic mice exhibit more repetitive alternations than naïve mice when navigating syllable space.

**Figure. 1.**
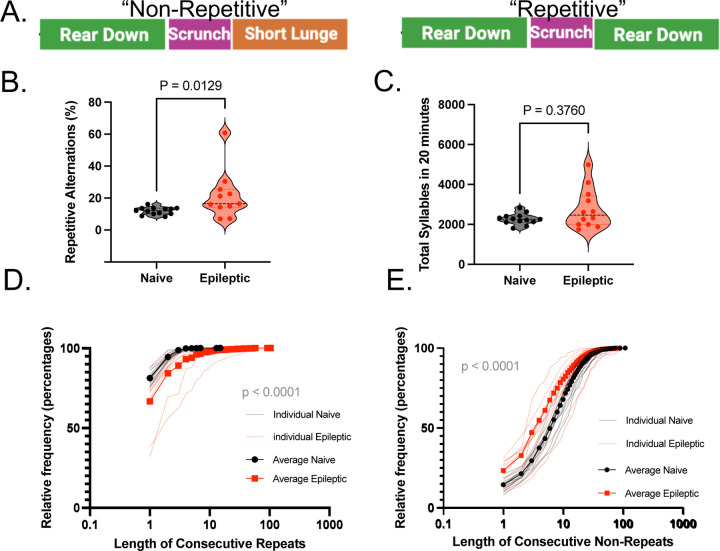
Epileptic mice have significantly more failed alternations than naïve mice. **(A)** Examples of non-repetitive or repetitive alteration using a three-syllable window**. (B)** Violin plots represent the percent repetitive alternations for naïve and epileptic mice and **(C)** total sequence length. Each point represents a mouse and statistics performed using the Mann-Whitney test. **(D)** Cumulative of the length of consecutive repetitive alternations for epileptic and naive mouse (Kolmogorov-Smirnov, D = 0.5071). **(E)** The length of consecutive non-repetitive alternations for each individual epileptic and naïve mouse was transformed into an average cumulative frequency distribution depicted in bold with the individual distributions represented by faint lines (K-S, D = 0.4903).

Previous work using MoSeq in epilepsy has focused on changes in syllable usage between epileptic, naïve and drug treated mice(25). Though each syllable on its own has little clinical relevance, we reasoned that when sequenced together, we may be able to use concatenated syllables as behavioral motifs which may hold greater significance. Therefore, for each sequence we defined non-repetitive behaviors as “non-repetitive alternations”, such that for a behavioral motif consisting of 3 sequential syllables, the first and third syllable are not the same (e.g. “rear down”->”scrunch”->”short lunge”). A motif where the first and third syllable are the same was classified as a “repetitive alternations” (e.g. “rear down”->“scrunch”->”rear down”) (Fig 1A). We find that epileptic mice have a significantly higher percent of ‘repetitive alternations’ compare to naïve mice (Mann-Whitney test p = 0.0129) (Fig 1B). This was not the result of differences in total movement between the two groups (Mann-Whitney test, p = 0.3760) (Fig 1C).

We then tested whether the increase in repetitive alternations was the result of extended bouts of repetitive behavior, also known as perseveration. Indeed, epileptic mice have significantly longer strings of consecutive repeats compared to age-matched naïve mice: 75% of naïve mice exhibit lengths of 1 repeat or fewer, whereas 75% of epileptic mice exhibit repeats lengths of 6 or fewer (K-S Test, p < 0.0001, D = 0.5071) (Fig 1D). Therefore, epileptic mice are not only repeating behaviors more often, but they are also repeating behaviors for longer stretches of time. Epileptic mice also have significantly shorter strings of consecutive non-repetitive alternations compared to age-matched naïve mice (Fig 1E): 50% of naïve mice show a minimum of 5 non-repetitive alternations compared to 3 for epileptic mice (K-S Test, p < 0.0001, D = 0.4903). This suggest that the ability of epileptic mice to have non-repetitive alternations is frequently interrupted by a repetitious alternation.

### Epileptic mice exhibit altered transition networks that prioritize a few nodes with high connectivity.

**Figure 2.**
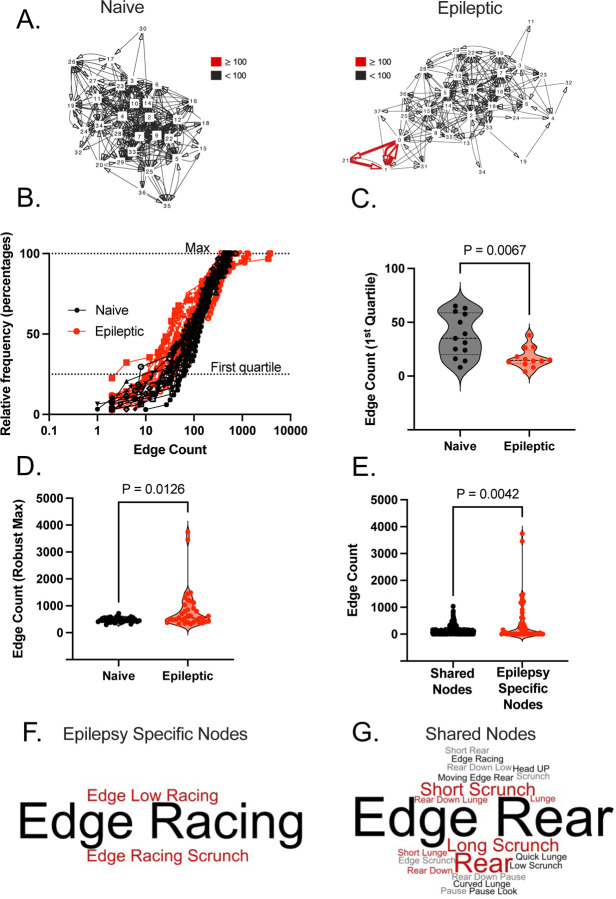
Epileptic mice exhibit abnormally connected networks with a racing phenotype. **(A)** Directed network diagrams of a single epileptic and naïve mouse where each node represents a ‘syllable’, and each edge represents a transition. Multiple transitions between nodes result in thicker and darker colored edges. **(B)** The individual node edge counts for each individual epileptic and naïve mouse were transformed into a cumulative frequency distribution using relative frequency (percentage). Violin plots represent the descriptive statistics from the **(C)** first quartile and **(D)** robust max of the frequency distribution with each point representing an individual mouse. Robust max was calculated using the top three values. Statistical analysis performed using the Mann-Whitney Test. **(E)** A violin plot represents the edge counts of nodes only present in epileptic mice compared to nodes shared between epileptic and naïve mice. Statistics calculated using K-S Test. **(F)** Word clouds created for the epilepsy specific nodes and the **(G)** nodes shared between epileptic and naïve mice. In both word clouds size indicates how often a node name is repeated in the node name list for a given group.

We projected the sequential transitions from one syllable to the next as a directed and weighted network. Each node represents a syllable and each edge represents a transition from one syllable to the next weighted by the frequency of that transition. Representative naïve and epileptic networks are shown in Fig 2A. The representative epileptic network shows many low-weight edges and a few high weight edges whereas the naïve network shows a range of medium weight edges indicating that some epileptic mice may have nodes with abnormally strong connectivity (see supplemental for all individual epileptic and naïve networks). One way to analyze network connectivity is using edge count, a metric of how connected a given node is to its neighbors. We took the individual node edge counts for each individual naïve and epileptic mouse and generated a cumulative frequency distribution (percentage) (Fig 2B). Analysis reveals that in the first quartile (representing nodes with lower edge counts) epileptic mice have significantly lower edge counts (Mann-Whitney Test, p = 0.0067) (Fig 2C) but at the robust max (representing nodes with high connectivity) epileptic mice have significantly higher edge counts than naïve mice (Mann-Whitney Test, p = 0.0126) (Fig 2D). This indicates that epileptic mice have sacrificed the integration of low connectivity nodes in favor of a few highly connected nodes.

To determine whether the aberrant connectivity of chronically epileptic mice was the result of new behavioral motifs or the increased usage of behavioral motifs found in naïve mice we analyzed edge count of the 7 epilepsy specific nodes compared to the 30 nodes shared between naïve and epileptic mice. The epilepsy specific nodes have significantly higher edge counts than the nodes shared with naïve mice (K-S Test, p = 0.0042) (Fig 2E). The behavior these nodes represent are related to ‘edge racing’ (Fig 2F) and racing more broadly which was not represented in the rest of the naïve shared syllables (Fig 2G). This indicates that the aberrant connectivity of the epileptic networks could be due to the emergence of a racing phenotype in epileptic mice.

### ‘Racing’ syllables are upregulated in epileptic mice 12 weeks post SE and persists as epilepsy progresses.

**Figure 3.**
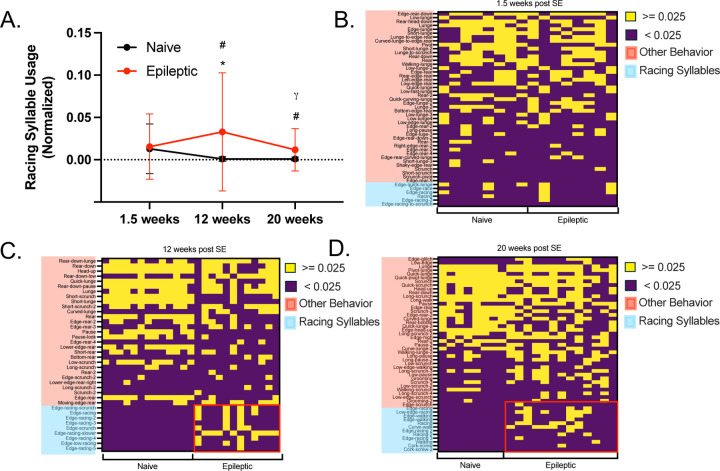
Racing syllables are upregulated in epileptic mice during chronic disease. **(A)** A summary line graph represents the racing syllable usage for naïve and epileptic mice 1.5-, 12-, and 20-weeks post status epilepticus. # indicates a significant difference between naïve and epileptic (q < 0.0001 at 12 weeks post SE, q = 0.0156 at 20 weeks post SE), * indicates a significant difference in epileptic racing syllable usage compared to 1.5 weeks post SE (q = 0.0067 at 12 weeks post SE). γ indicates a significant difference between epileptic mice 12 weeks post SE and mice 20 weeks post SE (q < 0.0001). **(B)(C)(D)** Binarized heatmaps for racing syllable usage were generated for each timepoint with each column representing an individual mouse. The threshold of 0.025 normalized racing syllable usage determined by the highest racing syllable usage of any 12 weeks post SE naïve mice. **(C)(D)** The red outlined box shows the area for racing syllable for epileptic mice 12- and 20-weeks post SE. All syllable usage values were normalized for each mouse. All statistics were performed using a two-way ANOVA test with BKY post hoc.

The presence of ‘racing’ in epilepsy specific syllables lead the investigation of racing behavior more broadly. We classified all the syllables, shared or epilepsy specific, based on the presence or absence of fast circular movement. To investigate whether racing syllable usage is prognostic of epilepsy we analyzed a 1.5-week post SE dataset for racing behavior prior to the emergence of chronic disease. We found racing is present in equal usage in both naïve and epileptic mice 1.5-weeks post SE (2-way ANOVA, q = 0.3531) (Fig 3A). In this 1.5-week post SE dataset all syllables were shared between epileptic and naïve mice indicating less of a divergence in behavioral phenotypes (Fig S1). At a later time point (12-weeks post SE) this racing phenotype significantly decreases in naïve mice (2-way ANOVA, q = 0.0315) but continues to increase in epileptic mice (2-way ANOVA, q = 0.0156) (Fig S1). Racing syllable usage remains elevated 20-weeks post SE (2-way ANOVA, q = 0.0156) (Fig 3A).

To view racing behavior at a more granular level we generated a binarized heat map of racing and non-racing syllable usage (Fig 3B–D). Naive mice 12 weeks post SE, have the lowest racing syllable usage; therefore, we set the threshold for binarization at the highest normalized racing syllable for aged naïve mice 12 weeks post SE, 0.025. At 1.5-weeks post SE we see no difference in the binarized matrix between naïve and epileptic mice (Fig 3B). However, at 12-weeks post SE every epileptic mouse shows usage of at least one racing syllable whereas no naïve mice show racing. Further, those epileptic mice 12 weeks post SE that use multiple racing syllables show a clear deficit in non-racing syllable usage (Mann-Whitney Test, p = 0.0061) (Fig S2). Once the mice have progressed to 20-weeks post SE, almost all but 2 epileptic mice still have 1 or more racing syllables (Fig 3D).

### MoSeq detects a shift to sedentary phenotype as epilepsy progresses from 12 to 20 weeks post SE.

**Figure 4.**
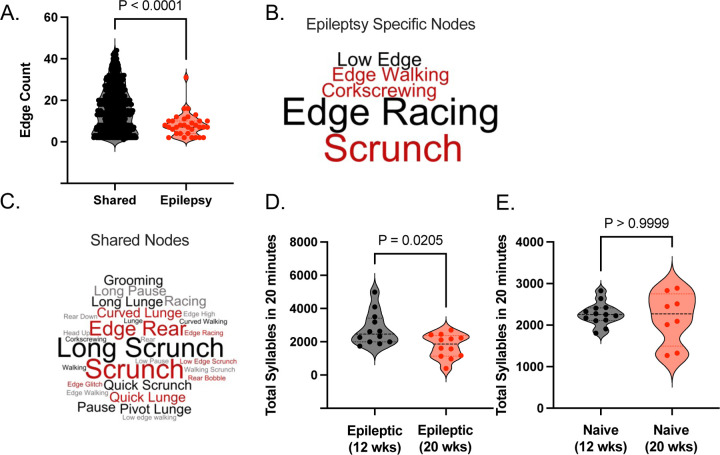
As epilepsy progresses to 20 weeks post SE syllables shift to a more sedentary profile. **(A)** Violine plots shows the reduction in edge count for epilepsy specific nodes at 20 weeks post status epilepticus. (Kolmogorov-Smirnov test, p < 0.0001). Each point represents an epilepsy specific nodes’ edge count. Depictions of word clouds showing the behaviors in **(B)** epilepsy specific nodes and **(C)** shared nodes for 20 weeks post SE. **(D)** Violin plots shows that sequence lengths are shorter from 12 to 20 weeks post status epilepticus **(E)** while naïve mouse lengths are statistically the same for each timepoint. Each point represents a mouse. Statistics done using a Kolmogorov-Smirnov test.

We find that as epilepsy progresses mice become increasingly sedentary and at 36-weeks post SE, they cannot participate in traditional Y maze tests (Table S1). To study this further, we generated a directed network (as described in figure 3) for mice 20 weeks post SE. Once again there are a selection of 6 nodes that only appear in the epileptic networks. These epilepsy specific nodes have lower edge counts compared to nodes shared with naïve mice (K-S Test, p < 0.0001) (Fig 4A). This contrasts with 12-weeks post SE when epilepsy specific nodes were aberrantly connected (Fig 2D). The behavior of epilepsy specific nodes 20 weeks post SE highlights ‘racing’ behavior and ‘scrunch’ (Fig 4B) while the shared nodes show a range of behaviors including ‘edge-rear’ and ‘long-scrunch’ (Fig 4C). The prominence of the ‘scrunch’ behavior in the epilepsy specific nodes 20-weeks post SE links disease and sedentary behaviors in a way not seen 12-weeks post SE. To further investigate the differences between epileptic mice 12- and 20-weeks post SE we analyzed the total sequence length for epileptic mice at each time point. We show that 20-week post SE epileptic mice have significantly shorter sequence lengths than mice 12-weeks post SE (Mann-Whitney Test, p = 0.0205) (Fig 4D). To test that this was due to disease and not aging per se we compared total sequence length between age match naïve mice at each time point and found no difference in total sequence length (Mann-Whitney Test, p > 0.9999) (Fig 4E). This indicates that 20 weeks post SE epileptic mice move through less total syllable space than 12-week post SE mice, potentially due to a sedentary effect.

### Network analysis reveals increased fragility of behavioral networks as epilepsy progresses.

**Figure 5.**
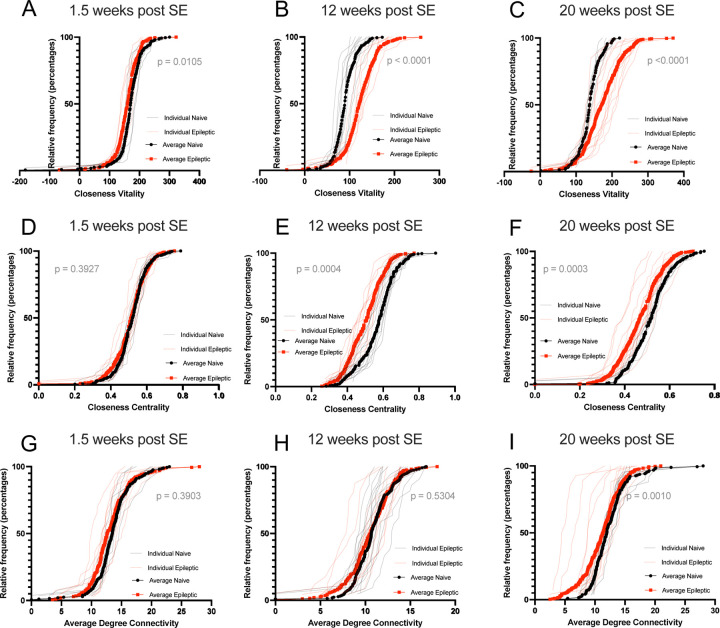
As epilepsy progresses behavioral network fragility increases. The closeness vitality for each node for all naïve and epileptic mice was transformed into cumulative frequency distributions for three different timepoints (1.5- **(A),** 12- **(B),** and 20- **(C)** weeks post SE) with the average distributions in bold and the individual distributions in the background. Statistical analysis was performed using the Kolmogorov-Smirnov test with a D statistic of 0.3275 **(A)**, 0.5799 **(B)**, and 0.5913 **(C)**. The closeness centrality for each edge count for all naïve and epileptic mice was transformed into cumulative frequency distributions for three different timepoints (1.5- **(D),** 12- **(E),** and 20- **(F)** weeks post SE) with the average distributions in bold and the individual distributions in the background. Statistical analysis was performed using the Kolmogorov-Smirnov test with a D statistic of 0.1800 **(D)**, 0.5000 **(E)**, and 0.4500 **(F)**. The average degree connectivity for each node for each individual naïve and epileptic mouse was transformed cumulative frequency distributions for three different timepoints (1.5- **(G)**, 12- **(H)**, and 20- **(I)** weeks post SE) with the average distributions in bold and the individual distributions in the background. Statistical analysis was performed using the Kolmogorov-Smirnov test with a D statistic of 0.2292 **(G)**, 0.2386 **(H)**, and 0.5160 **(I)**.

To test whether aberrations in behavioral networks are exhibited early and persist throughout disease progression, we generated directed networks for individual mice at three different timepoints in epilepsy progression (1.5 weeks post SE, 12 weeks post SE, and 20 weeks post SE). We measured edge count, betweenness centrality, and average neighbor degree (Fig S2). Closeness vitality, average degree connectivity, and closeness centrality stood out as significantly perturbed measures. Closeness vitality refers to the change in sum of edge lengths when a node is removed from the network and is a measure of network fragility i.e. how easily a network is perturbed when a node is removed. Comparison of the distributions revealed a slight decrease in closeness vitality at 1.5 weeks post SE (Fig5. A) (K-S Test, p = 0.0105, D = 0.3275). We see a larger increase in closeness vitality at 12 weeks post SE: 50% of the nodes in epileptic networks display a closeness vitality of up to 138 while 50% of the nodes in naive networks have a maximum closeness vitality of 108 (K-S Test p < 0.0001, D = 0.5799) (Fig 5B). This difference extends to 20-weeks post SE where 50% of the nodes in epileptic networks have a closeness vitality up to 219 as opposed to the 50% nodes of naïve networks with a max closeness vitality of 149 (K-S Test p<0.0001, D = 0.5913) (Fig5 C) suggesting that as epilepsy progresses the behavioral networks of mice are increasingly fragile.

We measured closeness centrality for each node of individual epileptic and naïve mice for each timepoint during disease progression plotting individual values as well as the average per condition (1.5-, 12-, and 20-weeks post SE) (Fig5 D–F). Closeness centrality refers to the reciprocal of the average shortest paths over all reachable nodes with the premise that increased closeness means increased centrality. Analysis revealed no significant difference in closeness centrality distribution between epileptic and naive mice at 1.5 weeks post SE (K-S Test, p = 0.3927, D = 0.1800) (Fig5 D). At 12-weeks post SE epileptic networks showed significantly decreased closeness centrality: 50% of the nodes in epileptic networks have a closeness centrality up to 0.547 whereas 50% of the nodes in naïve networks have a closeness centrality of up to 0.607 (12 weeks: K-S Test, p = 0.0004, D = 0.5000) (Fig 5E). This trend extends to 20 weeks post SE where 50% of the nodes of epileptic networks max closeness centrality of up to 0.537 whereas age matched naïve mice have a closeness centrality of up to 0.544 (20 weeks: K-S Test, p = 0.0003, D = 0.4500) (Fig5 F) indicating that the networks of epileptic mice are less centralized with fewer nodes close to each other.

We measured average degree connectivity for each edge value in each naïve and epileptic network during disease progression and plotted the individual values as well as the averages (1.5-, 12-, and 20-weeks post SE) (Fig5 G–I). Average degree connectivity refers to the average edge count of nodes neighboring nodes with a given edge count. Increased average degree connectivity display a high level of connectivity overall. We observe no difference in average degree connectivity between epileptic and naïve mice at both 1.5- and 12-weeks post SE (1.5 weeks: K-S Test, p = 0.3903, D = 0.2292) (12 weeks: K-S test, p = 0.5304, D = 0.2386) (Fig5 G–H). However, as disease progresses to 20 weeks post SE epileptic mice display significantly reduced average degree connectivity compared to age matched naïve mice (K-S Test, p = 0.0010, D = 0.5160)(Fig5 I) indicating epileptic mice are more dispersed behavioral networks.

## Discussion

In this study we applied a novel network analysis approach to characterize mouse behavior in Moseq analysis of disease progression in the systemic kainic acid mouse model of epilepsy. Our analysis reveals that chronically epileptic mice engage in perseverative behaviors and have altered behavioral networks characterized by the emergence of epilepsy specific ‘racing syllables’. We performed this analysis at three different timepoints in disease (1.5-, 12-, and 20-weeks post SE) to discover differences in epilepsy specific behavioral phenotypes and behavioral networks as disease progresses. A sequence and network-based approach to MoSeq data in a disease framework yield results with relevance to comorbidity analysis.

### Sequence analysis reveals perseverative behaviors 12 weeks post SE.

Patient reports as well as clinical studies have shown a correlation between psychiatric comorbidities, patients’ overall stigmatization, and reduced quality of life(10). However, many behavioral assays for comorbidities, such as depression, anxiety, and compulsive behaviors, in rodent models can only interrogate limited outcomes. These approaches utilize discrete tests that measure the ability to perform a specific well-defined task, such as the sucrose preference test, elevated plus maze, and marble burying test. Prior work by the Soltesz lab has demonstrated the ability of MoSeq to differentiate between epileptic and naïve mice in both acquired and genetic mouse models of epilepsy based on syllable usage(25). We expand on this work by testing whether analysis of concatenated syllables for repetitive alternation can further differentiate naive and epileptic mice. We find that epileptic mice repeat behaviors more often and for longer consecutive stretches than age-matched naïve mice. Others have shown that beyond syllable usage, analysis of transitions from one syllable to the next can be useful(21). We posit that this approach holds increased clinical relevance. A mouse ‘scrunch’ may not translate to a patient with epilepsy, but the emergence of behavioral motifs, such as repetition, may be better mapped on to the phenotypes of disease. For instance, patients with epilepsy have higher rates of both automatisms, defined as brief, repetitive, unconscious behaviors(27,28). These automatisms are generally associated with ictal events in both temporal lobe epilepsy as well absence seizures(27,28). TLE patients also have increased rates of obsessive-compulsive disorder (OCD) (11–34.5%) compared to the general public (0–3%)(9,29). Both of these clinical readouts could be plausibly mapped onto the repetitive behavior observed in our MoSeq analysis (8).

### Epilepsy specific syllables perturb behavioral networks 12 weeks post SE

We show that epileptic mice have perturbed behavioral networks resulting from the emergence of epilepsy specific syllables. We find that epileptic behavioral networks, generated from the transitions between incoming and outgoing syllables, have a few nodes with edge counts much greater than those present in naïve mice (Fig 2C). The high edge counts often connect 2 or 3 nodes in the network meaning this is the results of 2 or 3 syllables repeating back and forth. Furthermore, a subset of these highly connected nodes are specific to epileptic mice and not present in any naïve animals. This indicates that the perseverative phenotype is the result of novel syllables being introduced into the network. We find that epilepsy specific syllables are 1) hyper connected and 2) are defined by a ‘racing’ and an ‘edge racing’ phenotype: mice sprint around the edge of the arena. This aligns with reported observations of kindled mice which use novel syllables that are not present in non-kindled mice in MoSeq recordings(25): the racing syllables we report match the novel syllables of ‘wild running’ seen in mice kindled 5 minutes before MoSeq recording(25,30).

### Racing syllable usage decays with age in naïve mice but persist in disease

We find that racing syllable usage is not significantly different between epileptic mice early in disease and age-matched naïve animals. However, whereas naïve mice show an age dependent decrease in racing syllable usage, epileptic mice persist in racing behavior.

This suggests that racing syllables are a poor biomarker of epilepsy early in disease. MoSeq was previously used to analyze changes in syllable usage during the first 4 weeks of disease in the intra-hippocampal kainic acid model(25). We expand our analysis of disease progression in the systemic kainic acid model of epilepsy to 5 months. Furthermore, we noticed that mice manifesting multiple racing syllables showed a lower usage of non-racing syllables (figure xxx). It is tempting to hypothesize that the racing behavior may be connected to hyperactivity in epileptic animals. Others studying hyperactivity in rats and ADHD in humans have shown a link between interictal spiking and behavioral alterations (31–33). Levetiracetam, an FDA approved anti-seizure medication, has been shown to successfully suppress interictal spiking in 63% of patients with ADHD with and without concurrent seizures(34). Of the patients with suppressed interictal spiking 59% showed behavioral improvements(34). Future work will test the hypothesis that racing behavior is the result of interictal epileptiform discharges.

### Epilepsy progression from 12- to 20 weeks post SE is characterized by the emergence of a sedentary phenotype.

We find that as mice progress from 12- to 20-weeks post SE, epilepsy specific syllables manifest a reduction in edge count, signaling less connectivity to the overall network. This change corresponds with the emergence of epilepsy specific scrunching behaviors alongside the preexisting racing syllables. We believe that this change is due to disease progression rather than aging per se because naïve mice maintain their total syllable sequence length from 12–20 weeks post SE while epileptic mice show a significant reduction. Conventional y-maze tests only reveal a sedentary transition at 36 weeks post SE (Table S1). These data highlight the ability of MoSeq, with its focus on spontaneous behavior, to detect a shift in mouse behavior earlier in disease progression compared to conventional behavioral assays.

### Increased behavioral network fragility in epileptic mice.

Network analysis of individual mice reveals that while epileptic behavioral networks are not significantly different from naive at 1.5 weeks post SE, by 12-and 20-weeks post SE epileptic networks have multiple increasingly divergent properties. For instance, chronically epileptic mice exhibit more ‘fragile’ behavioral networks: they are populated by nodes with increased closeness vitality. The *closeness vitality* of a node is the change in the sum of distances between all node pairs when excluding that node(35). Thus, removal of a high closeness vitality syllable in an epileptic network results as cascading effects, including the increased dispersion of the network. While we observe significant differences in closeness vitality at 1.5 weeks post SE, at 12-, and 20-weeks post SE the effect size increases with disease progression (Fig 5A–C). This suggests that the changes in closeness vitality between epileptic and naive mice are more robust as disease progresses. Additionally, as disease progresses from 1.5 weeks post SE to 12- and 20-weeks post SE epileptic networks show a decrease in closeness centrality – a measure of how tightly the nodes of a network are connected (35): networks with low closeness centrality nodes tend to be dispersed and loosely connected. This suggests that epileptic networks are increasingly dispersed as disease progresses. At the same time, we find that as disease progresses, epileptic behavioral networks populate with syllables of decreased average degree connectivity – a measure of how connected neighbors of a node are (35). This indicates that from 12 to 20 weeks post SE epileptic mice have less connected, and more dispersed networks. As nodes become less connected both by distance and edge connectivity, they can become vulnerable to perturbations, supporting our closeness vitality measures. Together, these results suggest that as disease progresses the network loses resilience, i.e. if a node is removed from the network that loss has a greater impact on the topology of the network.

In sum, we have tested the use of network and syllable sequence-based analysis during disease progression in a mouse model of acquired epilepsy. Our findings of both 1) increased repetitive behaviors and 2) increased racing in chronic epilepsy map on to clinical comorbidities present in epilepsy patients. We propose that this framework can be used in other mouse models of progressive neurological diseases to derive clinically relevant behavioral outputs.

## Supplementary Material

Supplement 1
