# Supplementary material for: Motion sequencing reveals hidden patterns of repetitive behavior in a mouse model of epilepsy
Source: bioRxiv. 2025 Dec 9:2025.12.04.692371. Preprint. [Version 1] doi: 10.64898/2025.12.04.692371 (PMC12713134; doi:10.64898/2025.12.04.692371)
Supplement: Supplement 1 [file media-1.pdf]

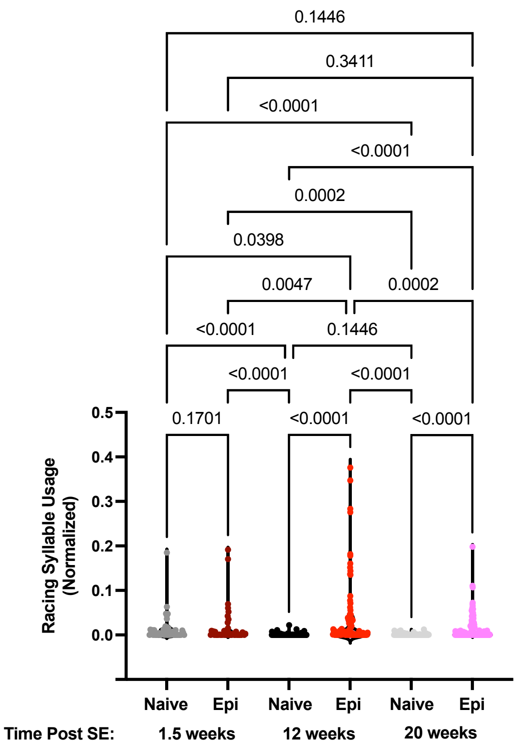

Fig. S1. Racing syllable usage increases as epilepsy progresses.
Violin plots depict the racing syllable usage for naïve and epileptic mice at three different timepoints (1.5-, 12-, and 20-weeks post SE). Each point represents a racing syllable. Statistical analysis conducted using the Kruskal-Wallis test with BKY post-hoc.

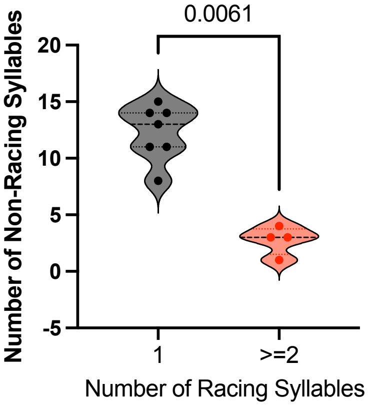

Fig. S2. Mice with multiple racing syllables display use less non-racing syllables.
Violin plots depict the number of non-racing syllables with a normalized usage over 0.025 for epileptic mice 12 weeks post SE. Each point represents a mouse. Statistical analysis conducted using the Mann-Whitney Test.

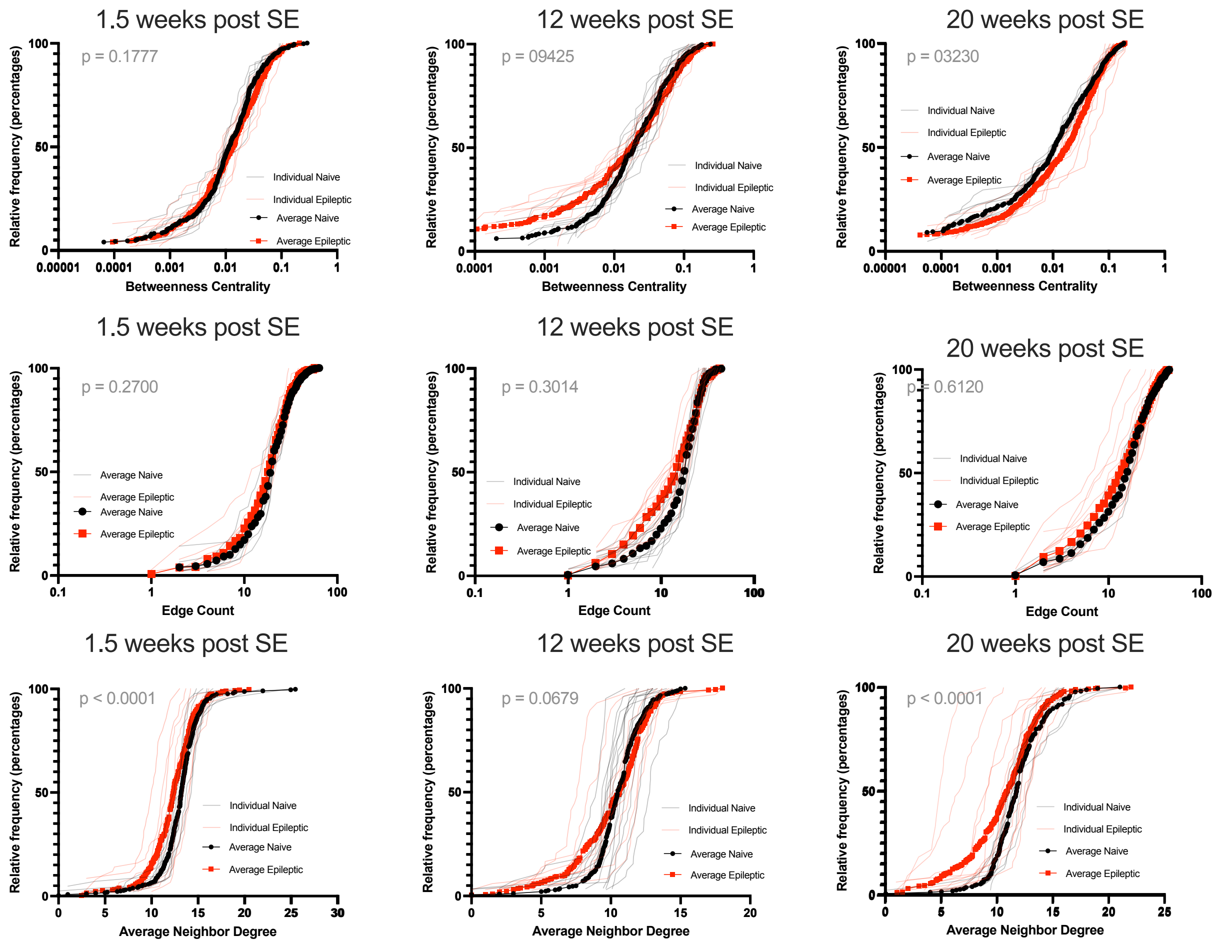

Fig. S3. Analysis of betweenness centrality, edge count, and average neighbor degree throughout disease progression. The betweenness centrality for each node for all naïve and epileptic mice was transformed into cumulative frequency distributions for three different timepoints (1.5- **(A),** 12- **(B),** and 20- **(C)** weeks post SE) with the average distributions in bold and the individual distributions in the background. Statistical analysis was performed using the Kolmogorov-Smirnov test with a D statistic of 0.1777 **(A)**, 0.1285 **(B)**, and 0.2042 **(C)**.The edge count for each node for each individual naïve and epileptic mouse was transformed cumulative frequency distributions for three different timepoints (1.5- **(D)**, 12- **(E)**, and 20- **(F)** weeks post SE) with the average distributions in bold and the individual distributions in the background. Statistical analysis was performed using the Kolmogorov-Smirnov test with a D statistic of 0.2000 **(D)**, 0.2361 **(E)**, and 0.1625 **(F)**. The average neighbor degree for each edge count for all naïve and epileptic mice was transformed into cumulative frequency distributions for three different timepoints (1.5- **(G)**, 12- **(H),** and 20- **(I)** weeks post SE) with the average distributions in bold and the individual distributions in the background. Statistical analysis was performed using the Kolmogorov-Smirnov test with a D statistic of 0.4600 **(G)**, 0.3160 **(H)**, and 0.5458 **(I)**.

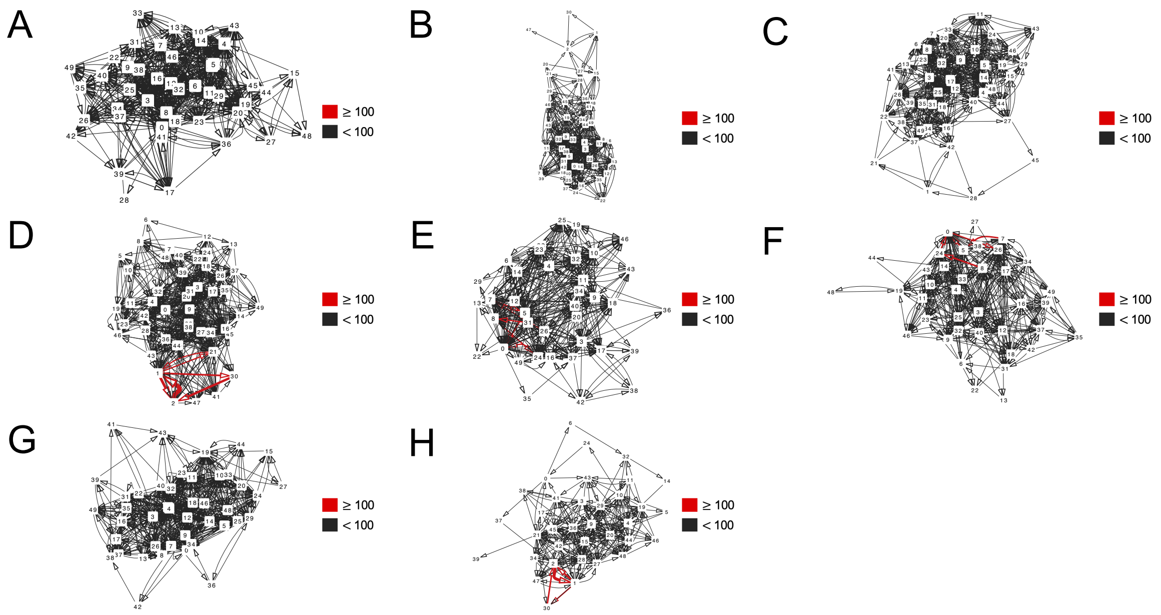

Fig. S4. 1.5 weeks post SE epileptic mice individual behavioral networks
Directed networks wherein each node represents a syllable, and each edge represents a transition. The edge is determined by the number of transitions. Edges with a frequency greater than or equal to 100 are colored red. Networks are ordered by mouse from **(A)** Epileptic 1 to **(H)** Epileptic 8.

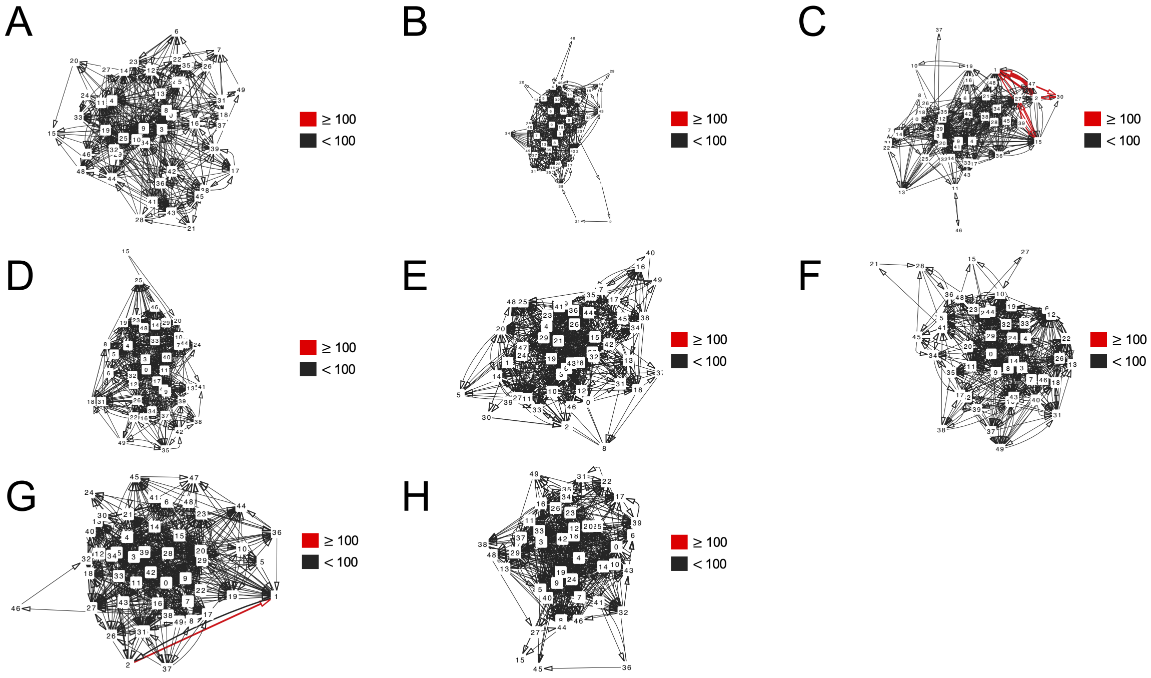

Fig. S5. 1.5 weeks post SE age-matched naive mice individual behavioral networks
Directed networks wherein each node represents a syllable, and each edge represents a transition. The edge is determined by the number of transitions. Edges with a frequency greater than or equal to 100 are colored red. Networks are ordered by mouse from **(A)** Naive 1 to **(H)** Naive 8.

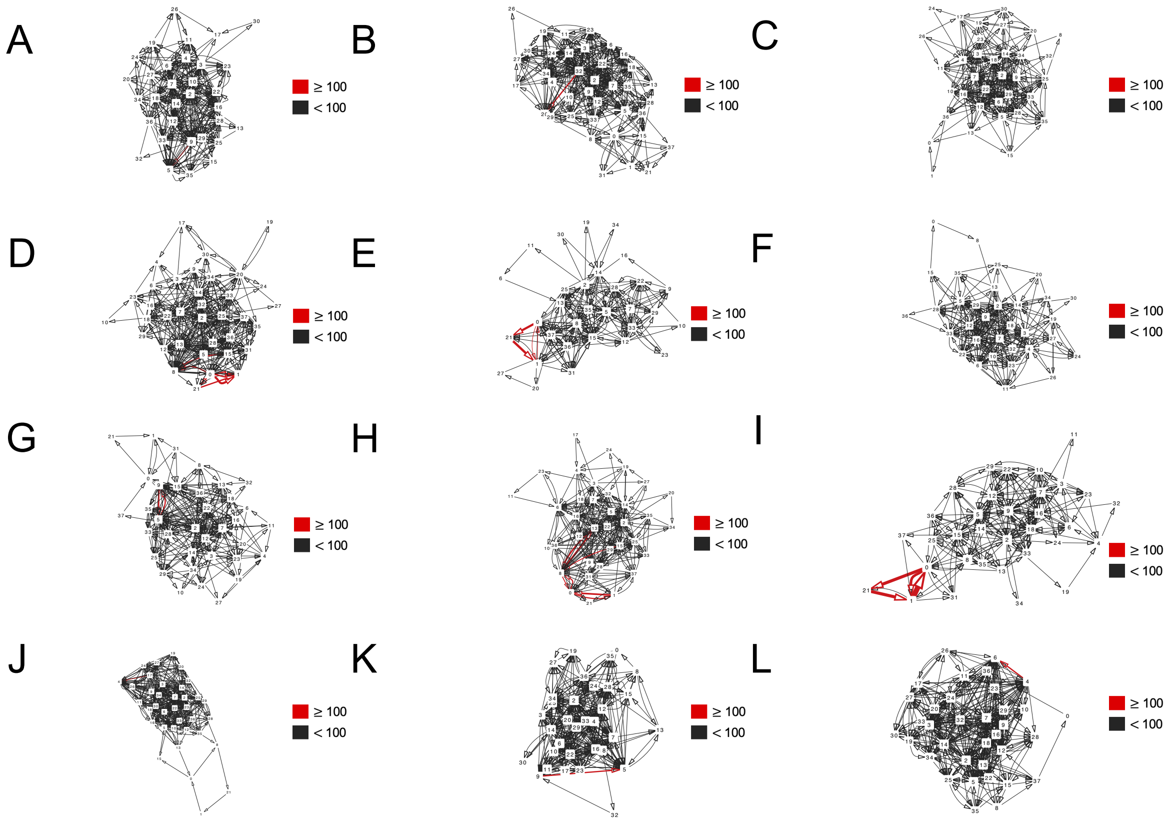

Fig. S6. 12 weeks post SE epileptic mice individual behavioral networks
Directed networks wherein each node represents a syllable, and each edge represents a transition. The edge is determined by the number of transitions. Edges with a frequency greater than or equal to 100 are colored red. Networks are ordered by mouse from **(A)** Epileptic 1 to **(L)** Epileptic 12.

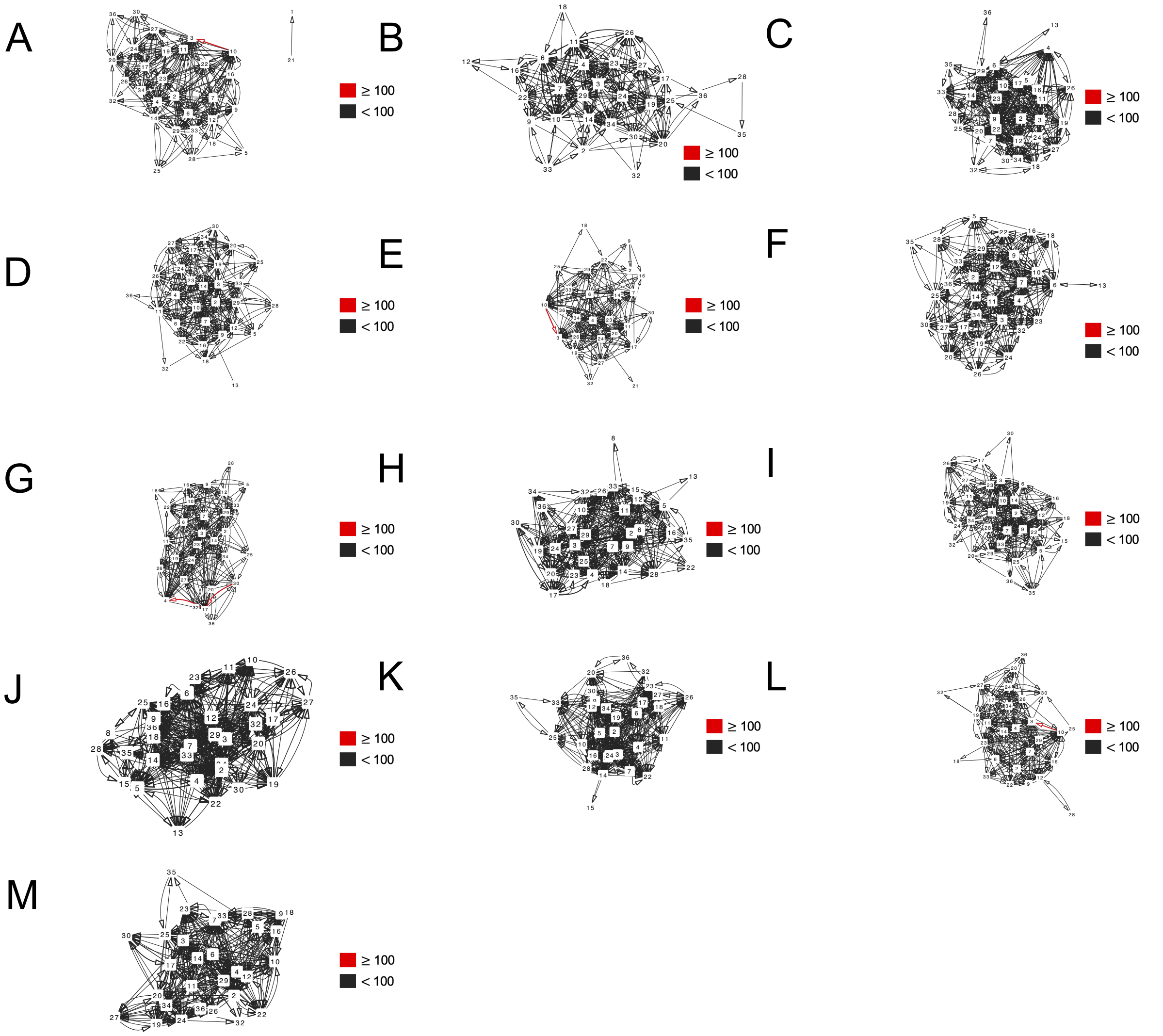

Fig. S7. 12 weeks post SE aged-matched naive mice individual behavioral networks Directed networks wherein each node represents a syllable, and each edge represents a transition. The edge is determined by the number of transitions. Edges with a frequency greater than or equal to 100 are colored red. Networks are ordered by mouse from **(A)** Naive 1 to **(M)** Naive 13.

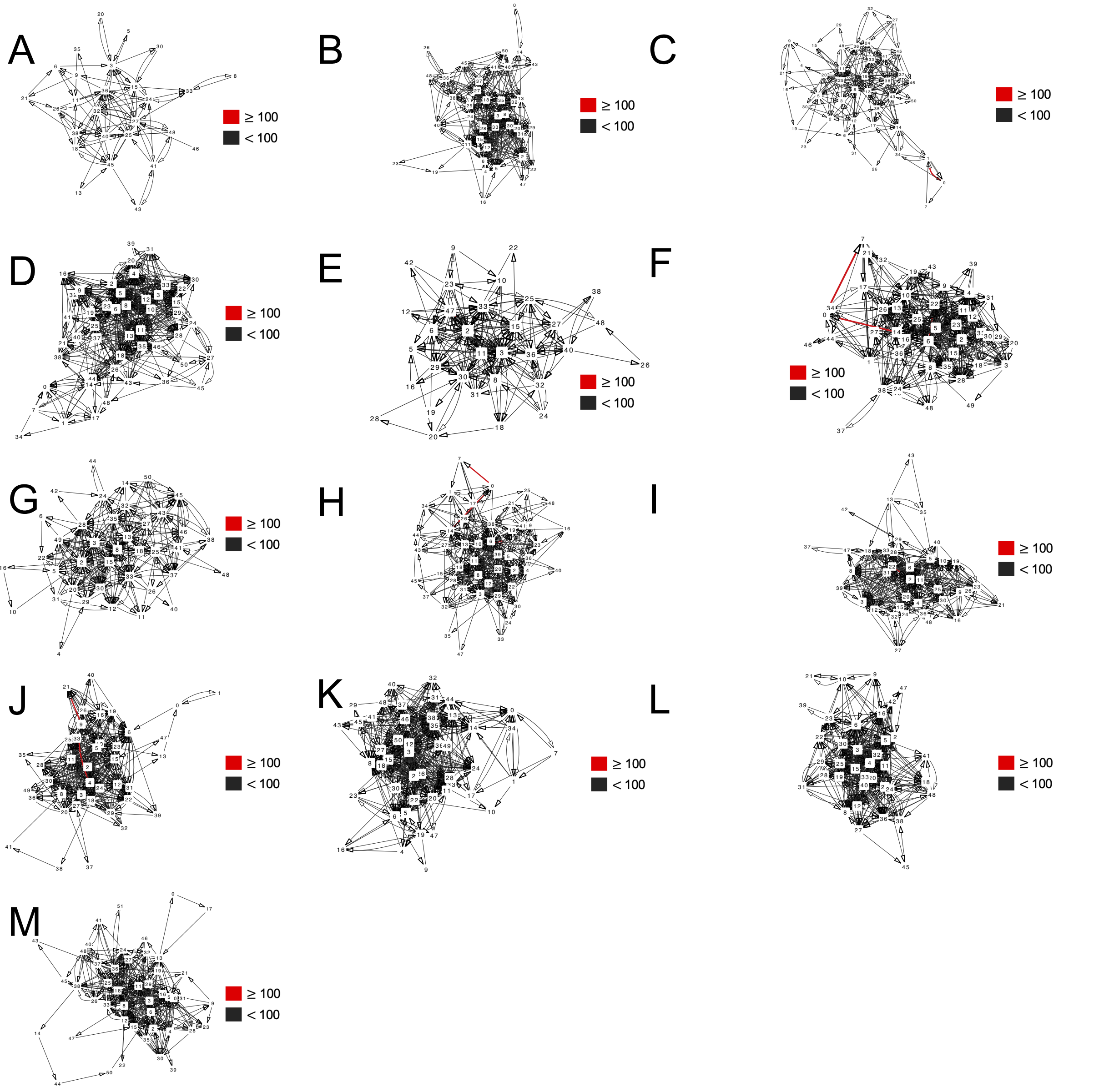

Fig. S8. 20 weeks post SE epileptic individual behavioral networks
Directed networks wherein each node represents a syllable, and each edge represents a transition. The edge is determined by the number of transitions. Edges with a frequency greater than or equal to 100 are colored red. Networks are ordered by mouse from **(A)** Epileptic 1 to **(M)** Epileptic 13.

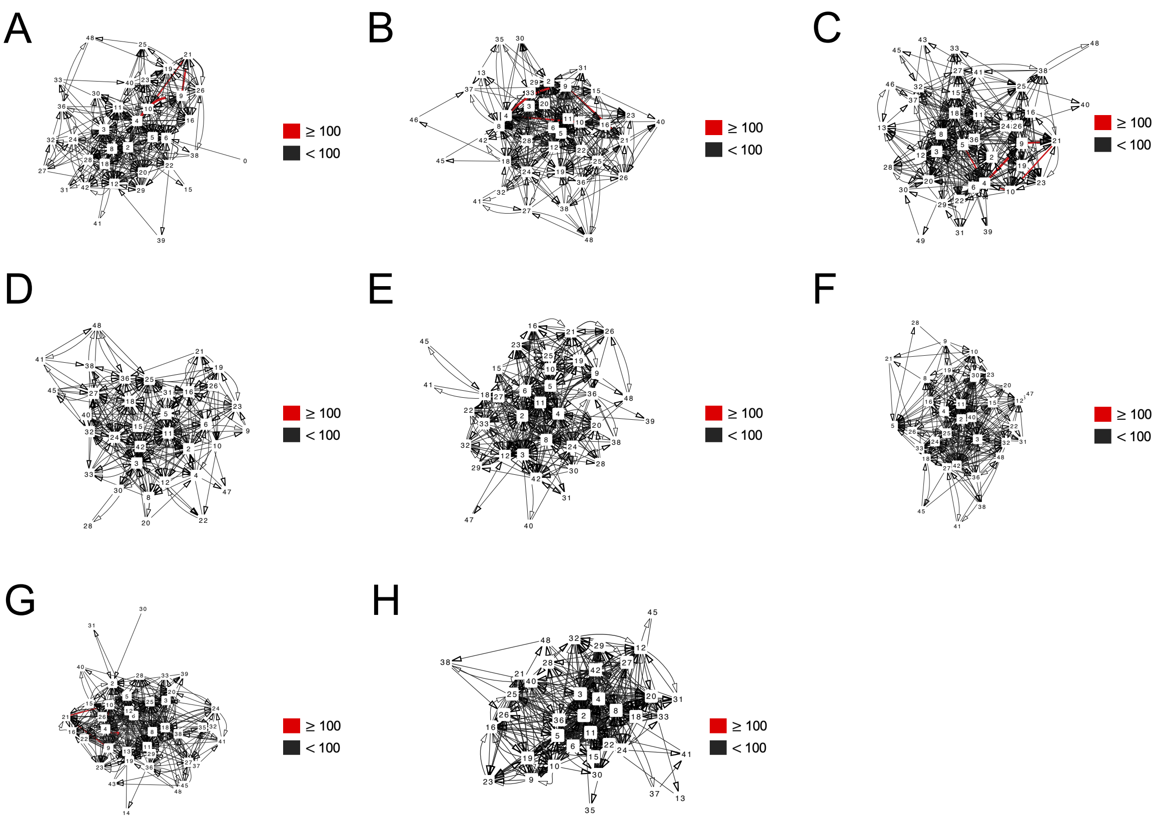

Fig. S9. 20 weeks post SE age-matched naive individual behavioral networks
Directed networks wherein each node represents a syllable, and each edge represents a transition. The edge is determined by the number of transitions. Edges with a frequency greater than or equal to 100 are colored red. Networks are ordered by mouse from **(A)** Naive 1 to **(H)** Naïve 8.

	Participated	Failed to Participate	Fisher's Exact Test p-value
**Age matched Naive**	9	0	
**Epileptic (34 weeks post SE)**	2	6	0.0023

Table S1. Participation in the spontaneous alternation Y maze test is impaired 34 weeks post SE in epileptic mice. Participation is classified as making 15 or more alternations in the Y maze in 8 minutes. Statistical analysis performed using the Fisher's exact test.
